# Genome-Wide Association Mapping of Macronutrient Mineral Accumulation in Wheat (*Triticum aestivum* L.) Grain

**DOI:** 10.3390/plants13243472

**Published:** 2024-12-11

**Authors:** Maha Aljabri, Mohamed El-Soda

**Affiliations:** 1 Department of Biology, Faculty of Science, Umm Al-Qura University, Makkah 21955, Saudi Arabia; 2 Department of Genetics, Faculty of Agriculture, Cairo University, Giza 12613, Egypt

**Keywords:** GWAM, macronutrient minerals, biofortification, wheat quality

## Abstract

The focus on increasing wheat (*Triticum aestivum* L.) grain yield at the expense of grain quality and nutrient accumulation can lead to shortages in macronutrient minerals, which are dangerous for human health. This is important, especially in nations where bread wheat is used in most daily dietary regimens. One efficient way to guarantee nutritional security is through biofortification. A genome-wide association mapping approach was used to investigate the genetic basis of the differences in macronutrient mineral accumulation in wheat grains. N, P, K, Na, Ca, and Mg concentrations were measured after a panel of 200 spring wheat advanced lines from the Wheat Association Mapping Initiative were cultivated in the field. The population exhibited a wide range of natural variations in macronutrient minerals. The minerals were found to have strong positive correlations except for magnesium, which had negative correlation patterns with N, P, and K. Furthermore, there were negative correlations between N and each of Ca and Na. Remarkably, genotypes with large yields contained moderate levels of critical metals. Of the 148 significant SNPs above −log10(*P*) = 3, 29 had −log10(*P*) values greater than 4. Four, one, and nineteen significant SNPs with a −log10(*P*) between 4 and 5.8 were associated with N and mapped on chromosomes 1A, 1B, and 1D, respectively. Three significant SNPs on chromosome A3 were associated with K. Two significant SNPs were associated with Ca and Na and mapped on chromosomes B3 and A4, respectively. Our findings offer crucial information about the genetic underpinnings of nutritional mineral concentration augmentation, which can guide future breeding research to enhance human nutrition.

## 1. Introduction

Wheat (*Triticum aestivum* L.) is the second most widely grown crop, with 790.42 million tons produced in 2024 [1]. Global consumption is expected to increase by 132 million tons by 2050 [2], necessitating more significant research into boosting output and quality. Wheat is essential for human health as it contains carbohydrates, proteins, and inorganic metals. Nonetheless, wheat grain has a naturally low nutrient content, including various minerals and vitamins essential to various biological processes. Maintaining mineral uptake and accumulation in wheat is critical for maximizing wheat growth, productivity, and nutritional quality [3,4], particularly for countries that rely heavily on wheat in their daily diet.

Similar to the depth of knowledge regarding the impacts of heavy metals [5,6,7,8,9], the overabundance of the necessary minerals has received attention [3,10,11]. While excessive uptake and accumulation of these minerals can interfere with growth, affect nutrient uptake, and cause mineral toxicity, nitrogen (N^+^), phosphorus (P^+^), potassium (K^+^), calcium (Ca^++^), and magnesium (Mg^++^) all play essential roles in various physiological processes [3,12,13,14].

Numerous biological substances, such as coenzymes, amino acids, nucleic acids, and chlorophyll, require N^+^. However, a high-N^+^ diet may cause adverse metabolic alterations that disrupt lipid and N metabolism in wheat, lowering grain filling [15,16]. In addition, P^+^ functions as a substrate in a range of physiological processes, including photosynthesis, respiration, signal transduction, and energy metabolism. It is a structural element of numerous biological molecules, including DNA, RNA, ATP, phospholipids, and carbohydrates [17]. On the other hand, excessive P^+^ consumption may inhibit meristematic activity and primary root growth, leading to deficiencies in other plant minerals such as iron (Fe^+++^) and zinc (Zn^++^) [18,19]. K^+^ improves photosynthesis, C^−^ and N^+^ metabolizing enzymes, and nitrate transport and assimilation, improving nitrogen utilization efficiency. However, high K^+^ consumption can hinder photosynthesis [20].

Na^+^ can accumulate in high quantities and be beneficial in replacing K^+^ in vacuolar osmoticum, where it acts as an osmotic agent. Except for a few halophytes, most plants do not require Na^+^ [21]. Ca^+^ deficit increases a plant’s susceptibility to biotic and abiotic stressors, as it plays a significant role in cell wall development, plant design, quality, and yield formation [22,23]. Mg^+^ is a cofactor for enzymes that support carbon fixation, metabolism, and chlorophyll [24]. Excessive magnesium intake can lead to a 54–67% decline in rice growth [25].

Developing stable varieties that balance vital mineral contents through traditional and modern plant breeding is a long-term method for maintaining mineral homeostasis [26,27]. However, this requires a better understanding of the genetic determinants controlling such quantitative features of mineral concentration [7,28]. Genome-wide association mapping (GWAM) effectively identifies genetic regions contributing to observed trait variation in a population. GWAM investigates genetic variations across the entire genome to discover genetic markers, such as single nucleotide polymorphisms (SNPs) statistically related to phenotypic variation [29].

Genomic prediction (GP) predicts phenotypes or breeding values of genotypes on unobserved individuals by connecting a set of markers to variability in cultivar phenotypes that have been observed [30]. In breeding programmes, GP relies on genome-wide marker data to forecast the breeding value of complex traits, offering an alternative to handle complex traits governed by numerous genes with minor impacts [7,30,31]. GP uses various techniques such as ridge-regression best linear unbiased prediction (rrBLUP), which enables effective prediction using unreplicated training data [32].

Our study was conducted to unravel the genetic architecture underlying five essential minerals and to identify candidate genes for these traits in spring wheat. In addition, we identified several genotypes with high contents of each of the measured minerals. Finally, we performed GP for the studied macronutrient minerals.

## 2. Results

### 2.1. Statistical Analysis

All traits exhibited a wide range of genetic variation (Table 1). The least observed variance was for Na, with minimum and maximum values of 0.06 and 0.12, respectively. Mg showed the second most minor variance, with a mean of 0.17, a minimum of 0.03, and a maximum of 0.38 mg kg^−1^. The P concentration ranged from 0.20 to 0.58, with an average of 0.34 mg kg^−1^. The minimum and the maximum of K were 0.21 and 0.62 mg kg^−1^, respectively. The most significant variations were observed for N and Ca, with a minimum of 0.58 and 0.11 and a maximum of 3.37 and 0.78 mg kg^−1^, respectively.

**Figure 1:**
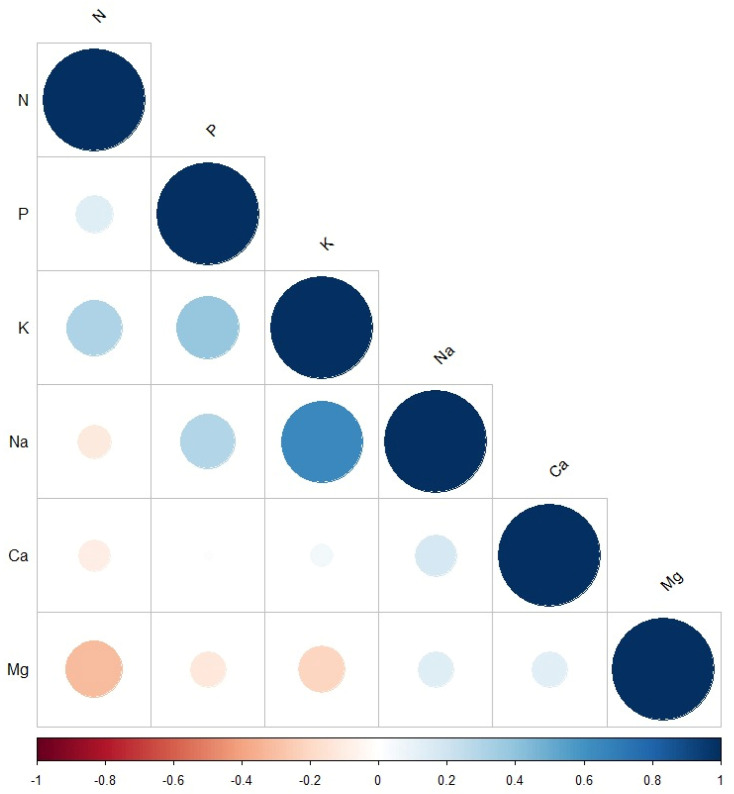
Correlation plot between the measured macroelement minerals: N, P, K, Na, Ca, and Mg. The size and the colour of the circles indicate the significance level. The blue and red indicate positive and negative correlations between the measured minerals.

The correlation plot (Figure 1) revealed a positive relationship between all evaluated parameters except between N and each of Na, Ca, and Mg. The strongest positive correlation was between Na and K, followed by the correlation between P and K. The strongest negative correlation was observed between N and Mg, followed by the correlation between K and Mg. No correlation was observed between P and Ca.

Checking the five high-yielding genotypes from Said et al. [33] for their macronutrient mineral concentrations revealed moderate concentrations of the six minerals (Table 2). We observed overlapping genotypes between the highest N, P, K, Na, Ca, and Mg contents. For example, genotype 80836 had the highest N content and the third-highest Ca content. Genotype 369673 showed the highest P and K contents.

### 2.2. Genome-Wide Association Mapping

**Figure 2:**
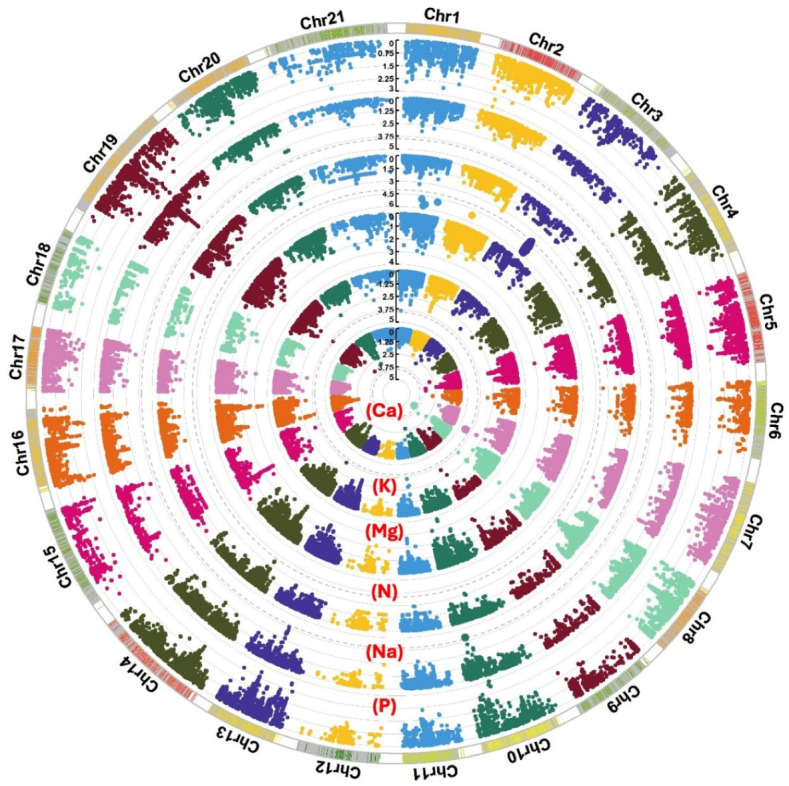
Circular Manhattan plots displaying significant SNPs associated with the wheat content of macronutrient minerals Ca, K, Mg, N, Na, and P at a −log10(*P*) value ≥ 3. Genome-wide association mapping was carried out for the 200 wheat genotypes using 26,814 SNPs distributed over the 21 wheat chromosomes. The significance level is denoted by dashed-grey circles.

A total of 148 significant SNPs, above −log10(*P*) = 3, were associated with the measured macroelement minerals (Figure 2 and Table S1), of which 29 SNPs were above −log10(*P*) = 4 (Table 3). For example, 24 SNPs between −log10(*P*) = 4 and 5.8 were associated with N and mapped on 1A, 1B, and 1D. Only one SNP, BS00021657_51, on 7A, was significantly linked to P. Among the 28 SNPs associated with K, one QTL harboured six SNPs linked to four genes at 177 cM on 3A. Three of the six SNPs were significant at −log10(*P*) = 4.3. Ten additional SNPs were linked to Na, four SNPs were mapped to 4A, and six SNPs were on 5A. One QTL sheltered 36 SNPs, significant at −log10(*P*) = 4.3, on 2B at 134 cM that were associated with Ca and contained 20 genes. A total of 11 SNPs showed a significant link to Mg, of which three were on 2B, three were on 4A, and five were on 6A.

### 2.3. Genomic Prediction

The predictability values for our wheat panel, which ranged from 0.036 to 0.14, were low. A prediction of 0.059 for N, 0.039 for P, 0.14 for K, 0.098 for Na, 0.078 for Ca, and 0.10 for Mg was obtained using the rrBLUP and a training population of 130 genotypes.

## 3. Discussion

The expansion of the world population places enormous pressure on agricultural systems to increase food production. However, this focus on quantity often ignores essential quality factors, including mineral imbalances [34]. It becomes obvious how nutritional imbalances interact, demonstrating how an excess or limited availability of one element can reduce the intake of other essential nutrients, ultimately affecting plant health and development [35,36]. Therefore, a balanced approach considering mineral buildup is required to overcome this difficulty.

Exploring natural phenotypic variation in wheat is the first step in boosting the levels of essential macronutrients in wheat grains using genomic breeding techniques [3,7]. We found significant natural variation in the grain concentrations of N, P, K, Na, Ca, and Mg using our panel of 200 advanced spring wheat lines, consistent with previous findings [3,5].

Choosing high-yielding genotypes with acceptable amounts of mineral accumulation is the foundation of biofortification [5,7,27,37]. We examined a variety of approaches to determine which genotypes performed best. First, we examined the five most productive genotypes from the previous study [33] that used the same population. Those genotypes showed moderate nutritional values of N, P, K, Ca, and Mg and low Na contents. Interestingly, those genotypes had some of the lowest Ni and Cd contents and some of the highest Mn, Fe, Cu, and Zn contents [7]. Our results imply that those genotypes could be good candidates for further breeding efforts to increase their content of the macronutrient minerals. The pyramiding of proteins and micro- and macronutrients into high-yielding wheat genotypes can alleviate malnutrition, affecting about one-third of the global population [38].

According to an earlier investigation [39], increasing nitrogen availability in the soil via a high input level enhanced wheat grain N content. At 238 kg ha^−1^ of ammonium nitrate (33.5% N) employed in our study, the five most productive genotypes [33] had a moderate level of N content. Instead, another five genotypes showed double N content. N is necessary to structure many biological molecules, including coenzymes, amino acids, and proteins. Therefore, it can indicate protein content (PC) using nitrogen-to-protein conversion factors [40,41,42]. Using wheat grains in dietary regimes can meet up to 25% of the protein needs [43]. However, breeding wheat to enhance grain yield and PC is still tricky due to the established negative correlation between the two characteristics [44,45]. Our results showed similar trends: high-yielding genotypes showed moderate N content, and genotypes with the highest seed N contents had low grain yield. Therefore, more breeding efforts should investigate how to improve wheat nutritional quality, particularly protein content.

High-quality wheat genome sequencing provides the base for identifying genetic loci and genes controlling wheat macronutrient content [46]. We used 200 advanced spring wheat lines from the WAMI population, genotyped with 26,814 SNPs [47,48], and mapped 148 significant SNPs above −log10(*P*) = 3, of which 29 SNPs were above −log10(*P*) = 4. The GAWM approach benefits from ancestral recombination, giving a higher resolution than the traditional QTL mapping using biparental populations [29]. The threshold −log10(*P*) = 3 is commonly used in wheat research [3,7]. However, the population structure of the GWAM population results in a high probability of false-positive associations. Therefore, we discuss SNPs with −log10(*P*) ≥ 4, multiple significant SNPs at the same position, or those that collocate with earlier findings.

A QTL associated with Ca content harboured 32 SNPs on Chr. 2B at 134 cM and two more SNPs at 136 and 141 cM. This QTL collocated with the SNP Kukri_c900_1334 [3] and the SNP IACX6292 [12] associated with Ca content. Another colocation observed for Ca was the SNP RAC875_rep_c78007_349, which was mapped here on Chr. 7B and collocated with wsnp_BE445506B_Ta_2_1 and wsnp_BE445506B_Ta_2_4 [12]. The SNP Kukri_c9895_1325 on Chr. 3B was linked to Ca and collocated with a previous SNP associated with Ca accumulation in wheat [14]. One more SNP, wsnp_Ku_c28104_38042857, was reported to be associated with Ca on Chr. 7A [49], collocated with our SNP wsnp_Ex_rep_c67593_66232317 mapped here.

The SNP BS00068508_51 mapped on Chr. 3A was linked to K [3], collocated with the six SNPs mapped here for the same trait (Table 3 and Table S1). One more SNP, BS00047691_51, was reported to be associated with P on Chr. 7A [49], collocated with our SNP BS00021657_51 mapped here for P. Four SNPs, wsnp_Ex_c34597_42879693, RFL_Contig6053_3082, and wsnp_Ex_c2236_4189774, on Chr. 6A and wsnp_Ex_c34597_42879718 on chromosome 6B were associated with Mg. The four SNPs were reported earlier to be significantly associated with Fe, Cu, and Cd using the same population [7]. We checked the correlation between the Mg and the three minerals, which revealed significant positive correlations of 0.6, 0.4, and 0.5, respectively, indicating the possible pleiotropic effects of the four SNPs on Mg, Fe, Cu, and Cd.

The significant positive correlations between the measured minerals reflect the presence of shared genetic mechanisms, known as pleiotropic effects, underlying the accumulation of these nutrients in wheat grains. Similar correlation trends have been previously reported in bread wheat [3,50]. In contrast, the significant negative correlations indicate possible antagonistic pleiotropic effects [51], critical in breeding programmes as breeding for one trait negatively impacts others. For example, negative correlations were observed between Mg and each of N, P, and K. Similar to our results, Alomari et al. [3] found a negative correlation between Mg and K and a moderate positive correlation between Mg and Ca. However, contradicting correlations were observed between Mg and P.

Generally, GP is a viable strategy for improving complicated features, like macronutrient metal content, mainly when studying a sizable germplasm panel with many markers. In line with earlier findings for wheat landraces evaluated in Afghanistan [52], both requirements enable more precise estimations of low breeding values for the tested metals. An individual is anticipated to perform below the reference population’s average for the trait under evaluation if their genetic breeding value is negative. Although GP values should range from 0 to 1, negative values are commonly reported. They can be explained by numerous factors, including a systematic bias of the correlation coefficient, as when the expected accuracy is low, negative prediction accuracy may be an artefact of the mathematical procedures used to calculate correlation coefficients. In addition, negative GP values can be due to statistical aberrations or remote QTL effects [53,54].

Our study offers valuable insights into the phenotypic diversity of the macronutrient minerals in the WAMI mapping population. For example, we observed that the five most high-yielding genotypes of the population have moderate concentrations of the six studied minerals. In contrast, genotypes with high seed N contents showed low grain yield. Therefore, pyramiding is required for high-yielding genotypes to increase their nutritional values. More studies are necessary to better understand the connections between the various mineral components in wheat and offer more specific knowledge that can result in practical breeding to address nutritional issues. The natural variation in the macronutrient minerals within the population facilitates GWAM investigations. Our results revealed 148 SNP markers with significant effects on the accumulation of micronutrient minerals in wheat grains. These data can be used in marker-assisted breeding programmes to improve wheat nutritional quality. In addition, it is necessary to validate those candidate genes or use genome editing technologies to edit new cultivars.

## 4. Materials and Methods

### 4.1. Plant Materials and Soil Conditions

We used 200 advanced spring wheat lines from the Wheat Association Mapping Initiative (WAMI) population and genotyped with 26,814 SNPs from the International Maize and Wheat Improvement Center (CIMMYT) [47,48]. The field study was carried out at the Agriculture Farm of Sohag University, Egypt. The soil texture is composed of two layers: sandy clay loam at depths ranging from 0 to 30 cm and sandy loam soil from 30 to 45 cm. The essential relevant chemical characteristics of the experimental soil are shown in Table 4.

The experimental setup used a randomized block design with three replications. Each block of a 2 m row with a 0.10 m plant spacing represented a genotype. NPK fertilization was as follows: 238 kg ha^−1^ of ammonium nitrate (33.5% N), 75 kg ha^−1^ of calcium superphosphate (15.5% P2O), and 58 kg ha−1 of potassium sulphate (48% K2O).

### 4.2. Mineral Measurements

A combination of concentrated HNO_3_ and HCLO_4_ (10:4) was added to 0.5 g of crushed wheat seeds in the digesting vessel, which was then sealed and cooked in a water bath at 80 °C for 120 min. The solution was cooled to room temperature and filtered through Whatman No. 1 filter paper into a volumetric flask containing 50 mL of double-distilled deionized water. N, P, K, Na, Ca, and Mg contents were determined in the extracts using an atomic absorption spectrophotometer (AAS) [55], LAAS-A11 (Labtron Equipment Ltd, Camberley, UK).

### 4.3. Statistical Analysis, Genome-Wide Association Mapping, and Genomic Prediction

Statistical analysis was performed using R for Windows version 4.1.2, with the R tool corrplot used to create frequency distributions.

Using the mixed linear model (MLM), kinship matrix, and principal component, the TASSEL program, version 5.0 [56], was used to map SNP markers associated with mineral contents.

GP was assessed using the R software package ridge-regression best linear unbiased prediction (rrBLUP) [32]. The 200 investigated genotypes underwent fivefold cross-validations, and the training sets consisted of 130 randomly selected genotypes. Following 100 iterations, the mean correlation accuracy was determined based on the correlation between the observed and predicted values, which validated the prediction ability computation.

### 4.4. Candidate Genes Identification

We retrieved the flanking sequences of the significant SNPs [57] and then identified the candidate genes using the GrainGenes BLAST service (https://wheat.pw.usda.gov/blast/, accessed on 28 February 2022), and the wheat IWGSC RefSeq v2.1 [58]. We used the KnetMiner gene discovery tool [59] (https://knetminer.org, accessed on 28 February 2022), to find large-genome-scale knowledge graphs and show intriguing subgraphs of relevant information regarding gene biology and functions, gene networks, and phenotypes.

## Figures and Tables

**Table 1:** Population performance of the measured macronutrient minerals: nitrogen (N), phosphorus (P), potassium (K), calcium (Ca), and magnesium (Mg). Minimum (min), maximum (max), and mean values in mg kg^−1^.

Macronutrient Minerals (mg·kg^−1^)	Min	Max	Mean	Standard Error
N	0.58	3.37	1.44	0.0304
P	0.20	0.58	0.34	0.0045
K	0.21	0.62	0.41	0.0031
Na	0.06	0.12	0.09	0.0007
Ca	0.11	0.78	0.31	0.0083
Mg	0.03	0.38	0.17	0.0050

**Table 2:** Macronutrient mineral contents in mg kg^−1^ for genotypes with the highest yield [33]: N, P, K, Na, Ca, and Mg concentrations. GY represents grain yield data from Said et al. [33].

Genotype	Macronutrient Minerals						GY	Category
	N	P	K	Na	Ca	Mg		
393392	1.18	0.37	0.38	0.086	0.27	0.23	25.01	High yield
1706327	1.29	0.31	0.42	0.095	0.34	0.16	26.32	
346403	1.06	0.36	0.34	0.076	0.19	0.15	26.94	
3597332	1.12	0.32	0.39	0.075	0.31	0.13	25.00	
294568	1.39	0.33	0.42	0.096	0.20	0.09	25.32	
80836	3.37	0.39	0.45	0.088	0.69	0.09	21.35	High N content
85599	3.06	0.34	0.44	0.088	0.17	0.14	16.90	
610288	2.94	0.34	0.4	0.065	0.25	0.07	14.98	
68315	2.90	0.45	0.46	0.088	0.20	0.07	12.60	
295261	2.90	0.37	0.48	0.010	0.30	0.06	18.63	
369673	1.88	0.58	0.62	NA	0.31	0.19	15.39	High P content
450975	2.86	0.53	0.44	0.069	0.15	0.08	18.84	
3669874	1.64	0.51	0.5	0.110	0.63	0.16	16.49	
1558746	1.31	0.51	0.45	0.105	0.44	0.18	15.87	
3586080	2.06	0.49	0.54	NA	0.29	0.25	23.30	
1812971	1.47	0.49	0.47	0.110	0.37	0.17	14.27	
369673	1.88	0.58	0.62	NA	0.31	0.19	15.39	High K content
3586080	2.06	0.49	0.54	NA	0.29	0.25	23.30	
3669874	1.64	0.51	0.50	0.110	0.63	0.16	16.49	
295261	2.90	0.37	0.48	0.100	0.30	0.06	18.63	
4318107	1.33	0.3	0.48	0.118	0.36	0.18	16.05	
4835640	NA	0.29	0.48	0.075	0.21	0.33	13.99	
4318107	1.33	0.30	0.48	0.113	0.36	0.18	16.05	High Na content
3600263	1.49	0.42	0.46	0.113	0.29	0.06	18.64	
4320047	1.39	0.30	0.46	0.113	0.24	0.20	13.06	
3669874	1.64	0.51	0.50	0.110	0.63	0.16	16.49	
1812971	1.47	0.49	0.47	0.110	0.37	0.17	14.27	
82710	1.51	0.48	0.43	0.110	0.41	0.22	13.57	
85587	1.37	0.30	0.35	0.086	0.78	0.15	19.97	High Ca content
41868	2.94	0.30	0.44	0.069	0.74	NA	12.59	
2244167	1.11	0.30	0.39	0.086	0.73	0.17	14.91	
3617481	1.14	0.29	0.41	0.094	0.69	0.18	17.37	
80836	3.37	0.39	0.45	0.088	0.69	0.09	21.35	
86005	1.47	0.35	0.37	0.095	0.30	0.38	13.75	High Mg content
126306	1.33	0.26	0.36	0.090	0.49	0.35	18.41	
640876	1.49	0.40	0.38	0.090	0.29	0.34	17.13	
88701	1.06	0.28	0.33	0.081	0.28	0.33	12.92	
41372	1.37	0.28	0.40	0.086	0.22	0.32	14.30	

**Table 3:** Selected significant SNPs associated with macronutrient mineral contents. The positions (Pos) in centimorgan on chromosomes (Chr.) are indicated. The −log10(*P*) values indicate the significance value. The explained phenotypic variance (R^2^) and the effect of each allele are given. Names of candidate genes are presented according to the second version of the International Wheat Genome Sequencing (IWGS2).

Trait	Marker	Chr	Pos	−LOG10 (*P*)	R^2^	Allele (Alternate)	Effect	Gene Name in RefSeq_v2
N	RAC875_c32452_55	1A	86	4.928	0.11	C(T)	−0.41	TraesCS1A03G0802700LC
	Excalibur_c8396_396		94	5.686	0.15	A(C)	−0.71	TraesCS1A03G0838000
	Excalibur_c8301_1555		101	5.501	0.12	A(G)	0.45	TraesCS1A03G0846000
	RAC875_c51346_99		144	4.971	0.11	C(T)	0.43	NA
	Excalibur_c3596_144	1B	86	5.228	0.11	A(G)	0.43	TraesCS1B03G0941800
	Ra_c1211_1656	1D	103	4.869	0.10	C(T)	0.41	TraesCS1D03G0769500
	BS00028216_51		104	4.596	0.10	A(C)	−0.39	NA
	IAAV6873		104	4.645	0.10	A(T)	0.41	TraesCS1D03G0772200
	wsnp_Ra_c17989_26960545		104	4.508	0.10	C(T)	0.38	TraesCS1D03G0786200
	Excalibur_c53900_86		104	4.157	0.09	C(T)	−0.37	TraesCS1D03G0781300
	Kukri_c12183_262		105	4.928	0.11	C(T)	0.41	TraesCS1D03G0780300
	CAP7_c9557_164		105	4.257	0.09	C(T)	−0.38	TraesCS1D03G0789100
	Ra_c3045_1739		105	5.356	0.12	G(T)	0.44	TraesCS1D03G0778200
	RFL_Contig651_953		107	4.086	0.08	A(C)	−0.37	TraesCS1D03G0792900
	Ra_c11906_1618		107	5.656	0.12	A(G)	−0.46	TraesCS1D03G0791300
	Ra_c11906_1441		107	5.639	0.12	A(G)	−0.01	TraesCS1D03G0791300
	Ra_c15730_3403		111	5.639	0.12	A(G)	−0.46	TraesCS1D03G0806600
	wsnp_Ra_rep_c70864_68811253		111	4.880	0.10	A(G)	−0.41	TraesCS1D03G0805600
	wsnp_Ku_c26635_36605013		111	5.639	0.12	C(T)	0.46	TraesCS1D03G0803500
	RAC875_c55026_311		112	5.527	0.12	C(T)	0.45	TraesCS1D03G0811800
	wsnp_Ku_c40309_48558476		112	4.458	0.09	C(T)	0.41	TraesCS1D03G0813500
	wsnp_Ex_rep_c111610_93458148		113	5.685	0.13	C(T)	0.46	TraesCS1D03G0813500
	RFL_Contig4705_3207		113	4.770	0.11	C(T)	0.41	TraesCS1D03G0813600
	BS00063907_51		115	5.269	0.11	C(T)	0.43	TraesCS1D03G0816500
K	wsnp_Ex_c361_707953	3A	177	4.357	0.10	A(G)	0.03	TraesCS3A03G1209700
	BS00068508_51		177	4.310	0.09	A(G)	0.03	TraesCS3A03G1210000LC
	wsnp_Ex_c361_708712		177	4.369	0.10	C(T)	−0.03	TraesCS3A03G1209700
Ca	D_F5XZDLF01EEKO2_217	3B	35	4.189	0.09	A(C)	−239.52	TraesCS3B03G1455900
Na	Excalibur_c2023_345	4A	139	4.106	0.08	A(G)	73.05	TraesCS4A03G1146300

**Table 4:** Chemical characteristics of the experimental soil at depths ranging from 0 to 30 cm (sandy clay loam) and from 30 to 45 cm (sandy loam soil).

Properties	Depth (cm)	
	00–30	30–45
Soil pH	7.5	8.2
ECe (dS/m at 25 °C)	2.1	2.5
Available nitrogen (ppm)	50	20
Available phosphorus (ppm)	20	22
Available potassium (ppm)	69	62
CaCO_3_ %	3.5	4.1
Organic matter %	1.9	1.4

## Data Availability

All data are available in the manuscript or its Supplementary Materials.

## Supplementary Materials

The following supporting information can be downloaded at: https://www.mdpi.com/article/10.3390/plants13243472/s1. Table S1: Significant SNPs associated with measured traits with their predicted genes.
